# Learning to See the Vibration: A Neural Network for Vibration Frequency Prediction

**DOI:** 10.3390/s18082530

**Published:** 2018-08-02

**Authors:** Jiantao Liu, Xiaoxiang Yang

**Affiliations:** School of Mechanical Engineering and Automation, Fuzhou University, Fuzhou 350108, China; m150210008@fzu.edu.cn

**Keywords:** vibration measurement, frequency prediction, deep learning, convolutional neural network, photogrammetry, computer vison, non-contact measurement

## Abstract

Vibration measurement serves as the basis for various engineering practices such as natural frequency or resonant frequency estimation. As image acquisition devices become cheaper and faster, vibration measurement and frequency estimation through image sequence analysis continue to receive increasing attention. In the conventional photogrammetry and optical methods of frequency measurement, vibration signals are first extracted before implementing the vibration frequency analysis algorithm. In this work, we demonstrate that frequency prediction can be achieved using a single feed-forward convolutional neural network. The proposed method is verified using a vibration signal generator and excitation system, and the result compared with that of an industrial contact vibrometer in a real application. Our experimental results demonstrate that the proposed method can achieve acceptable prediction accuracy even in unfavorable field conditions.

## 1. Introduction

Structural vibration measurement is widely used in civil engineering, mechanical engineering, and other engineering practices. The frequency components can be estimated from the measured vibration signal; thus, the resonant frequency or the natural frequency can be determined from the signal. The estimation of the resonant frequency or natural frequency of a mechanical system is of great importance in many engineering applications. The resonant frequency and natural frequency can be used in monitoring the mechanical behavior of important structural components, detecting variations in the mechanical properties, design optimization, avoiding the occurrence of resonance disasters, and so forth.

Vibration measurement methods can be divided into two categories: contact measurement and non-contact measurement. Contact measurement uses contact sensors attached to the measurement target such as velocity transducer, strain gauges, and accelerometer. However, installing and deploying contact sensors is both time and labor intensive. Non-contact measurement utilizes certain types of electromagnetic radiation to transmit information, such as laser Doppler vibrometer (LDV) [1,2,3] and microwave interferometry techniques [4,5]. The use of cameras to record visible light to carry out non-contact vibration measurement (also called image-based or computer vison vibration measurement) has received significant attention in recent decades, such as in digital image correlation (DIC) [6,7,8], marker tracking [9,10,11,12], and target-less [13,14] image-based vibration measurement methods. In the conventional image-based methods, the vibration signals should be extracted first to determine the vibration frequency. In the DIC method, patterns must be manually applied to the target object surface. Then, the image processing algorithm is implemented to track variations in the projected [15,16] or printed pattern [6] on the surface for correlation analysis of the vibration signals. Then, algorithms such as FFT are used to analyze the frequency components and finally determine the resonant frequency. Marker tracking also requires an optical target such as LED light [10] or marker [17] printed or mounted on the target surface, while the image algorithm and signal analysis algorithm are implemented consecutively. In the target-less method, the image processing algorithm must also be applied first to analyze and track the intrinsic features of the target object surface [13,18,19], and an algorithm is then implemented to determine the frequency spectrum. As discussed above, the present image-based non-contact vibration frequency measurement method requires a complex image algorithm for vibration signal extraction, as well as a signal analysis algorithm to estimate the vibration frequency, which requires the use of a substantial amount of computational resources. This makes it difficult to deploy conventional image-based non-contact methods in real applications.

In this work, we do not explicitly analyze and extract vibration signals from the image sequence; rather, we propose a convolutional neural network (CNN) trained with generated artificial signals, and utilize the learned features to discriminate the pixel brightness variation in the time domain for vibration frequency prediction. Furthermore, down sampling, de-noising, or other signal enhancement algorithms are not required. The proposed method predicts the vibration frequency at pixel level or the statistical result of pixels in the region of interest (ROI) using the original raw noisy brightness signals of each pixel and additional image processing or signal processing algorithms are not required. Verification tests were conducted using a vibration signal generator and excitation system. Further laboratory and field experiments were conducted to compare the performance of the proposed method with that of an industrial vibrometer. The limitations of this study and challenges in a real application are then discussed.

## 2. Methods

This section describes the learning of deep features for vibration frequency prediction using our CNN model. First, the proposed artificial neural network architecture is introduced. Then, we will describe how the artificial signals were generated in the dataset preparation stage and the training procedure. The entire pipeline of the proposed method is presented at the end of the section.

### 2.1. Network Architecture

**Figure 1:**
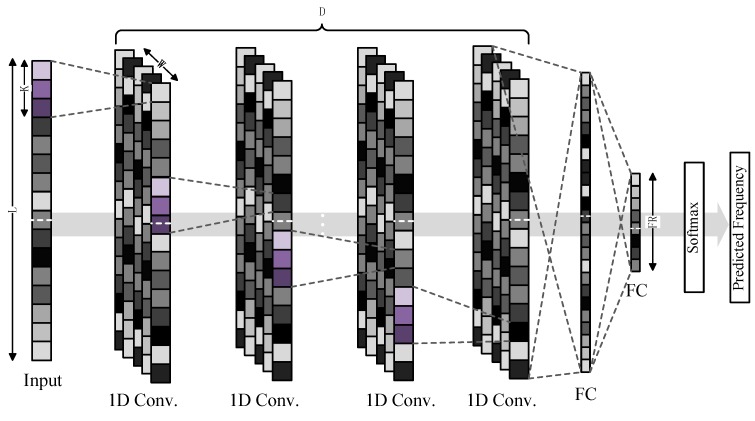
Convolutional neural network architecture of proposed method.

To discriminate the brightness signals of the raw noisy pixels, we treated the vibration frequency prediction as a multi-class classification problem, utilizing the learned deep features to classify input information into different classes, which are the values of the predicted frequencies. Figure 1 shows the proposed neural network architecture. The proposed network is composed of D one-dimensional convolution layers and two fully connected layers; each convolution layer has W outputs and is followed by a batch normalization (BN) [20] layer, a max pooling layer [21], and activated by a rectified linear unit (ReLU) [22] activation function. We used K × 1 kernels for each convolution layer. The input one-dimensional information length L is the product of the sampling rate and video clip duration. The fully connected layers connected all input neural nodes, which function as high-level reasoning in the artificial neural network captured and correlated features activated by different parts of the input signal. Since most parameters are collected in the fully connected layer, overfitting can easily occur. The dropout [23] layer, which is a regularization method that randomly sets several neural nodes to zero, is followed by the first fully connected layer to effectively prevent overfitting, and all the fully connected layers also consist of an ReLU. The fully connected layers connected all activations into the frequency range (FR) output. The FR is defined as the frequency range multiplied by the reciprocal of the prediction precision. For example, if the measurement frequency range is 0–200 Hz and the precision is 0.2 Hz, the FR value is 200 × 1/0.2, which is equal to 1000. FR outputs are fed into a softmax function to yield a probability distribution over every FR class.

In general, we optimize for a deeper (larger D) and thinner (smaller W) network architecture. We varied D from 2 up to 6, and for every convolution layer, we used a kernel with kernel size K in the range 3–13. Furthermore, we padded the input of every convolution layer by (K-1)/2 pixels with 0 on both sides to maintain the spatial dimensions along the depth.

### 2.2. Dataset Preparation

The proposed CNN was trained with large amount of purely artificial data. Both the training data and testing data were generated by an artificial random process.

**Figure 2:**
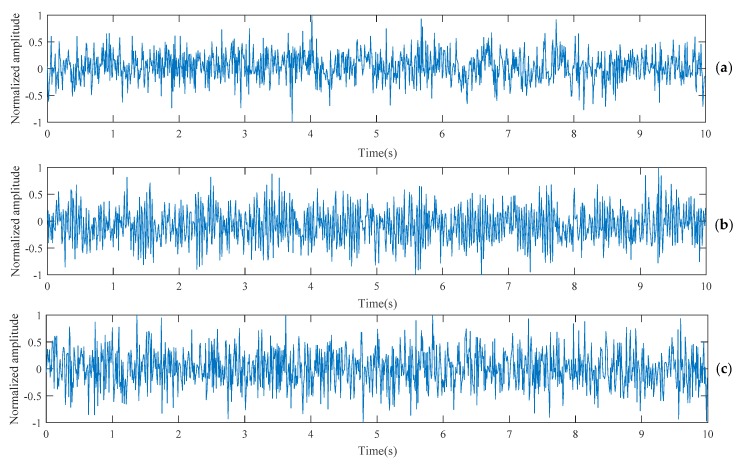
Examples of artificial signals: (**a**) Artificial signal of 5.6 Hz vibration frequency with random noise added; (**b**) Artificial signal of 26.5 Hz vibration frequency with random noise added; (**c**) Artificial signal of 46.8 Hz vibration frequency with random noise added.

To simulate real vibration signals, we used a simple artificial vibration signal generator to generate vibration signals with a specific vibration frequency. Artificial noise random sample from a Gaussian distribution with zero mean and varying standard deviation was added to the artificial signals to improve generalization of the CNN and avoid overfitting. The standard deviation of the artificial noise was set to vary between 0.6 and 2.1, which will achieve the best result after testing from 0 to 10. Detail of the artificial signal generating function is given in Equation (1). *T* is the discrete time value from zero to the duration of the artificial signal with an even step of $\frac{1}{FS},$ where *FS* is the sampling rate, f is the frequency of the artificial signal, and A is the signal amplitude. Examples of generated artificial signals are shown in Figure 2.

(1) A=sin(2πf×T)+Noise

For each frequency precision step, we generated approximately 3000 or even more artificial signals and the corresponding ground truth vibration frequency value was labelled with each artificial signal. For instance, if FR is 100, there would be at least 300,000 artificial signals generated for the training process.

### 2.3. Training Procedure

At the training stage, we optimized the weights and bias parameters by minimizing the negative log likelihood loss over the entire training dataset. In all our experiments, we used the adaptive moment estimation (Adam) optimizer [24], which is a stochastic gradient descent-based optimizer and maintained the adaptive estimate of the first and second order moment of the gradient. We used a learning rate of $1\times 10^{-4}$, and did not decrease the learning rate while using a larger batch size of 4096, which was inspired by Smith et al. [25]. The training was carried out on Pytorch [26] with an nVidia GTX1080ti, which normally requires 12 h to 1 day to converge to a good solution.

### 2.4. Implementation Pipeline

**Figure 3:**
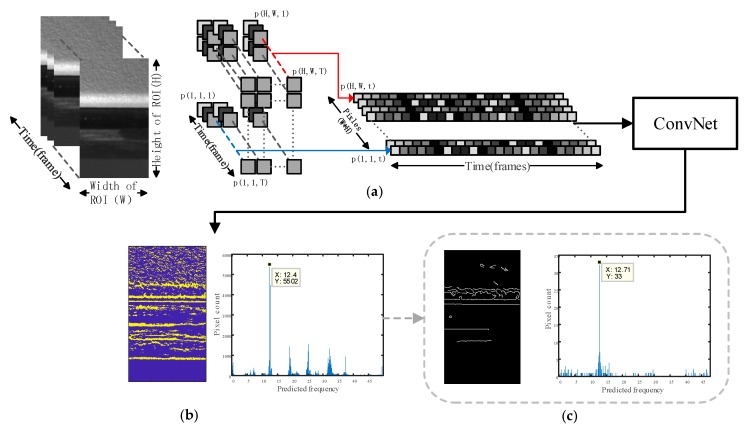
Implementation pipeline of the proposed method: (**a**) Read in the ROI video as image sequence and save as separate pixel brightness variation signals, then feed in the ConvNet; (**b**) Network output prediction result visualization; (**c**) Optional edge enhancement operation.

The ROI for frequency prediction was selected in the input video; then, the cropped video of the ROI was read out as image sequences. The brightness values of every pixel of the image sequence in the time domain were saved as separate pixel brightness variation signals. As shown in Figure 3a, every pixel in the ROI is read out along the time dimension from the first frame to the end frame. If the width and height of the ROI are W and H, respectively, we will obtain W×H number of pixel signals along the time dimension. For instance, if we have a 500 fps 9×9 image sequence with duration of 5 s, we will obtain 81 (9×9) instances of input with length L equal to 2500 (500 Hz × 5 s). Then, the instances were directly fed into the trained CNN to yield the number of predicted vibration frequency as output. After obtaining the predicted vibration frequency of each pixel, we can generate a vibration frequency map corresponding to the original image spatial information as shown in the left plot in Figure 3b. A histogram of the predicted frequency distribution is plotted in Figure 3b, so that the result can be quantified for an overall frequency prediction. For the frequency corresponding to the maximum of the histogram, the pixels predicted around this frequency are highlighted for a better understanding of the prediction result, as shown in the middle of Figure 3b.

An optional operation can be implemented if the input video is recorded in an unfavorable condition such as if it contains extremely noisy signals, variations in the lighting condition, and camera shake. We described such operation as edge enhancement operation, that is, to perform Canny edge detection on the first frame of the image, and only take pixels at the edge into consideration in the statistical procedure when generating the histogram plot, while pixels outside the edge are ignored. Improved vibration frequency prediction result can be obtained because pixels around the image edge always have better contrast, which is very important in all photogrammetry methods.

### 2.5. Experimental Methods

Several experiments were conducted, including a verification test using the vibration test system, impact hammer excitation test in a controlled laboratory condition, and field experiment, similar to a practical application. An industrial camera with Aptina MT9P031 image sensor was used in all the experiments and the DongHua DH5906 industrial vibrometer was used for comparison of results obtained from the proposed method in laboratory and field experiments.

In all experiments presented in this paper, the camera sampling rate was set to 100 Hz, and 10 s of video was recorded for each experiment case. The input information length L was 1000 (100 Hz × 10 s), and the prediction FR value was 500 since the frequency range is 0–50 Hz and the precision is defined as 0.1 Hz. The experiments described in this section used a network with D = 4 layers (each with W = 10 and K = 11). Since we must test D from 2 to 6, W from 5 to 15, and K from 3 to 13, this configuration ensured that the network exhibited fast convergence to a usable model and acceptable prediction accuracy without many redundant parameters. The prediction accuracy did not benefit from a deeper network. Choosing W = 10 features per layer worked well, W = 15 is superfluous, while W = 5 will significantly decrease the convergence speed and prediction accuracy. K = 11 was found to be the best choice considering the accuracy and convergence speed. In the edge enhancement operation, the high and low thresholds for the Canny edge detection algorithm were set to values that would generate a clear edge for the target.

#### 2.5.1. Verification Test

**Figure 4:**
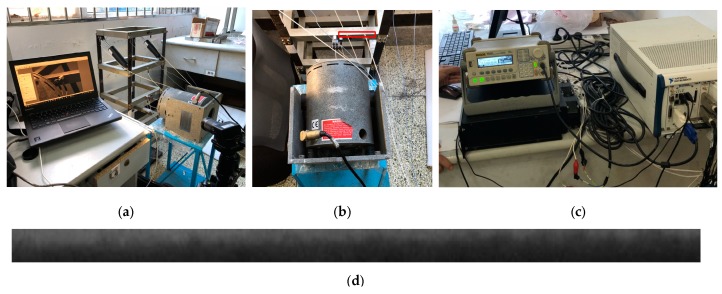
Experimental setup for verification test: (**a**) Camera, laptop, and steel structure; (**b**) MB Dynamics MODAL 50 exciter (ROI highlighted in red rectangle); (**c**) RIGOL DG1022 arbitrary waveform generator; (**d**) ROI for vibration frequency measurement.

An experiment was conducted to validate the proposed method using a vibration test system, which consisted of a RIGOL DG1022 arbitrary waveform generator, MB Dynamics MODAL 50 exciter, and SL500VCF amplifier. The experimental setup is shown in Figure 4.

Sine signals were generated every 5 Hz between 0 and 50 Hz; then, the signals passed through the amplifier with minimum gain and to the exciter. A modular steel structure was excited by the precisely controlled vibration signals, and a video of the measurement ROI in Figure 4d, which was set to the beam near the exciter with a resolution of 468 × 14 pixels, was recorded simultaneously. Then, the proposed method was applied to the recorded videos to determine the vibration frequency prediction of the measurement target ROI.

#### 2.5.2. Laboratory Experiment

**Figure 5:**
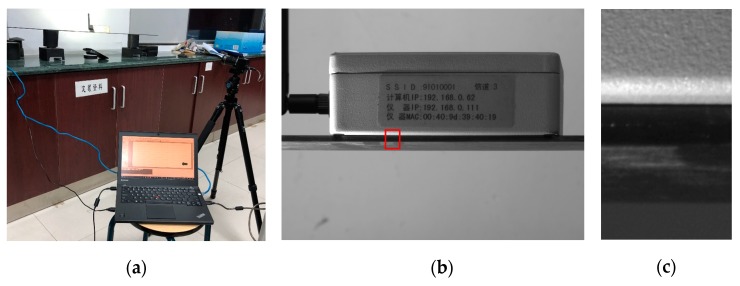
Laboratory experimental setup: (**a**) Camera, laptop, carbon plate, and vibrometer mounted at the midpoint; (**b**) Field of view of the camera (ROI highlighted in red rectangle); (**c**) ROI for vibration frequency measurement.

An experiment was conducted in a well-controlled laboratory environment to investigate the performance of the proposed method and the results were compared with those obtained using an industrial vibrometer. A carbon plate was struck on the left support point using an impact hammer as the excitation. The resulting vibrations of the carbon plate were captured by the vibrometer mounted at the midpoint of the simple-supported carbon plate and the sampling rate of the vibrometer was set to 100 Hz. The ROI of the camera was also set to the midpoint of the plate with a resolution of 176 × 304 pixels as shown in Figure 5c. An LED torch was used as additional light source for the target object, so that the camera can maintain a high sampling rate. The configuration is shown in Figure 5a.

The raw acceleration signal was transformed to the frequency domain using fast Fourier transform (FFT). The frequency components of the vibration signal were examined in the frequency domain and compared with the vibration frequency prediction result obtained from the proposed method.

#### 2.5.3. Field Experiment

To verify the applicability of the proposed method to practical engineering applications, a field experiment was conducted on the Wuyuan bridge, Xiamen. Since the bridge is a steel structure and was built on extremely salty marine environment, the bridge is surrounded by water vapor evaporating from sea; as the vapor cools, it condenses to fog with high salinity, which inevitably speeds up corrosion of the steel structural components. The local department of transportation actively monitors the dynamic behavior of bridge cables and other structural components. The vibration frequency is one of the most important parameters used in predicting the structure’s modal shape and tension force of structural components. A bridge cable was selected as the test object.

**Figure 6:**
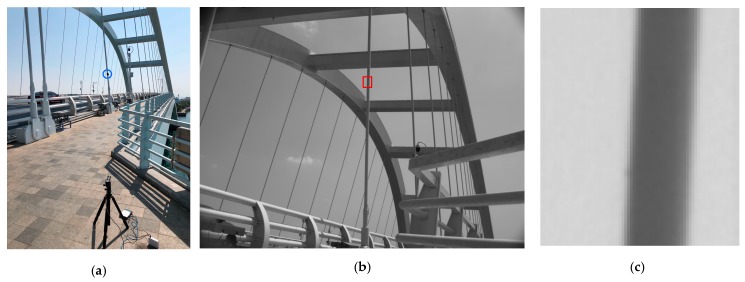
Field experimental setup: (**a**) Camera, receiver, vibrometer (highlighted in blue circle); (**b**) Field of view of the camera (ROI highlighted in red rectangle); (**c**) ROI for vibration frequency measurement.

In the field experiment, the bridge cable was excited by randomly passing vehicles at varying speeds, dissimilar pattern, and weight. The vibrometer was attached to the bridge cable with a sampling rate of 100 Hz, and a video of the ROI of the bridge cable was captured; the resolution of the ROI was 72 × 86 pixels as shown in Figure 6c. The frequency components of the bridge cable were obtained using FFT from the vibrometer acceleration time history. The proposed method was implemented on the video recorded in the field to determine the vibration frequency, which was compared with that of the vibrometer.

## 3. Results

### 3.1. Verification Test

**Figure 7:**
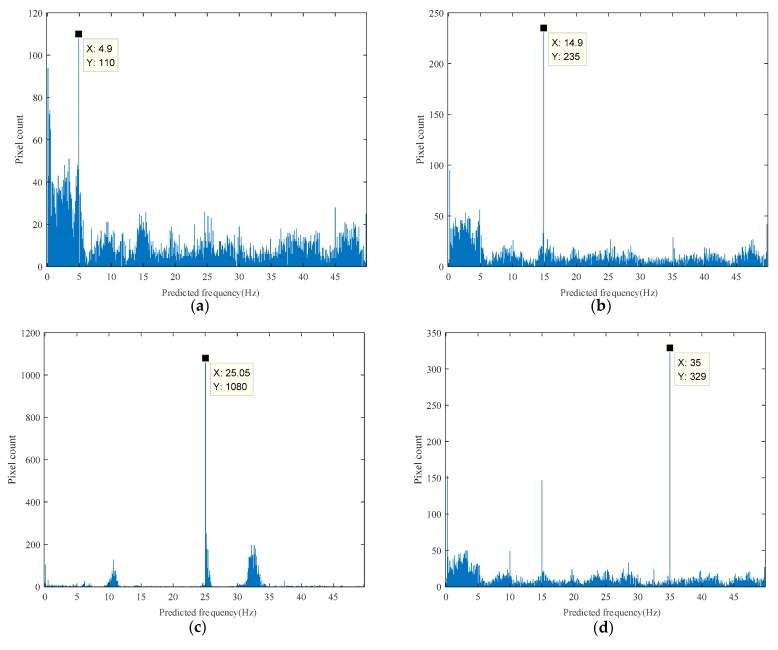
Four representative results of histogram of the predicted frequency distribution: (**a**) 5 Hz excitation result; (**b**) 15 Hz excitation result; (**c**) 25 Hz excitation result; (**d**) 35 Hz excitation result.

**Figure 8:**
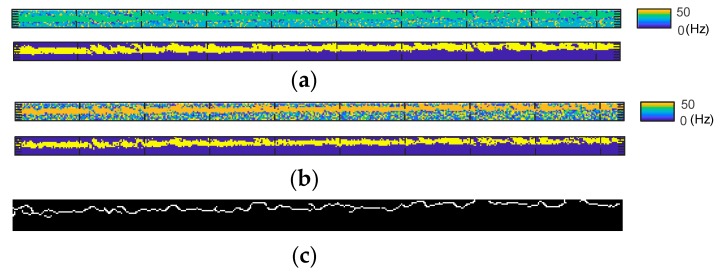
Two representative results and Canny edge detection algorithm result: (**a**) 30 Hz excitation result: prediction result map and pixels predicted around 30 Hz (maximum value of histogram) highlighted; (**b**) 40 Hz excitation result: prediction result map and pixels predicted around 40 Hz (maximum value of histogram) highlighted; (**c**) Pixels used in edge enhancement.

**Figure 9:**
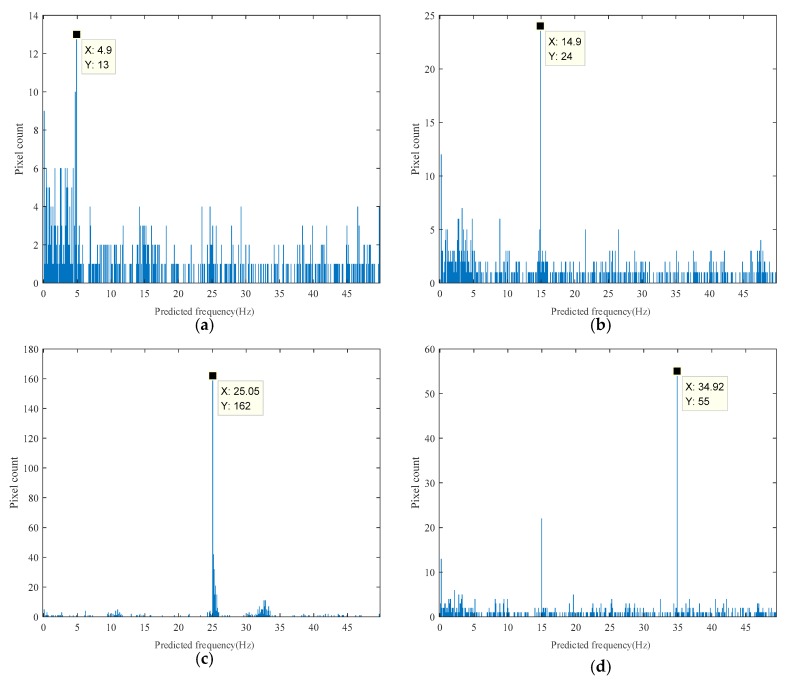
Four representative results of histogram of predicted frequency distribution after edge enhancement: (**a**) 5 Hz excitation result; (**b**) 15 Hz excitation result; (**c**) 25 Hz excitation result; (**d**) 35 Hz excitation result.

Figure 7 shows four results of histograms of the predicted frequency distribution with excitation frequencies of 5 Hz, 15 Hz, 25 Hz, and 35 Hz. The prediction visualized results for 30 Hz and 40 Hz are shown in Figure 8; the prediction results of every pixel mapped to its original spatial position on the input image and pixels predicted around the maximum value of the histogram were highlighted. Optional operation using the Canny edge detection algorithm of finding pixels on the edge is shown in Figure 8c; the high and low thresholds for the algorithm were set to 0.6 and 0.24, respectively in the normalized threshold range of 0 to 1. Figure 9 shows results of the corresponding excitation frequencies of 5 Hz, 15 Hz, 25 Hz, and 35 Hz obtained from edge enhancement in which pixels on the edge were taken into consideration in generating the histogram.

The results from nine different test scenarios are summarized in Table 1, including excitation with frequencies of 5 Hz, 10 Hz, 15 Hz, 20 Hz, 25 Hz, 30 Hz, 35 Hz, 40 Hz, and 45 Hz.

It can be observed from the above results that the proposed method successfully predicted the excitation frequency within an error range of 0.1 Hz. The optional edge enhancement operation is not necessary under such condition and did not improve the direct prediction results. Since the excitation is a standard clear sine wave without noise, the background and lighting conditions in the verification test are relatively stable so that noise in the captured videos was also low.

### 3.2. Laborotory Experiment

**Figure 10:**
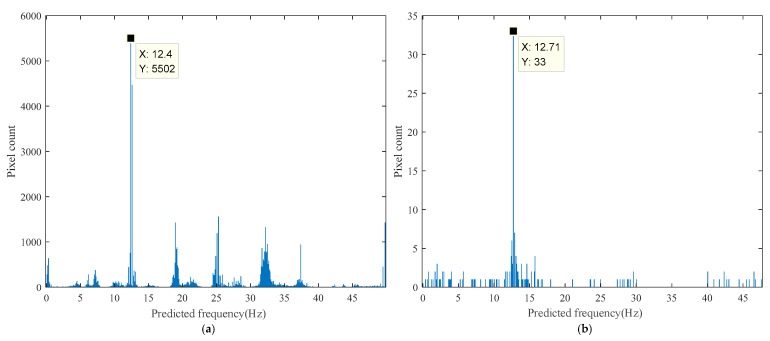
Laboratory experimental results of the proposed method: (**a**) Histogram of the predicted frequency distribution; (**b**) Histogram of the predicted frequency distribution after edge enhancement.

**Figure 11:**
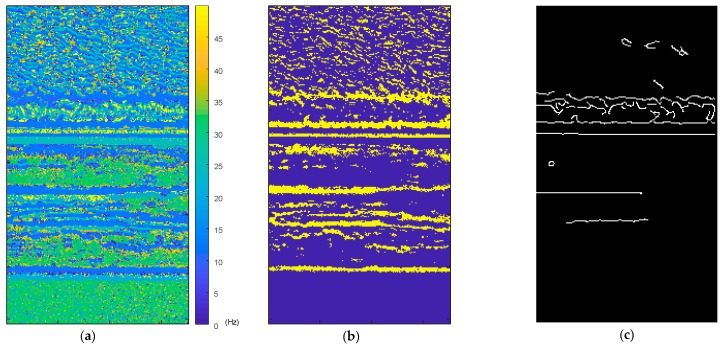
Laboratory experimental result visualization using the proposed method: (**a**) Prediction result map; (**b**) Pixels predicted at 12.4 Hz (maximum value of histogram) highlighted; (**c**) Pixels used in edge enhancement.

The histogram of the predicted frequency distribution obtained from the proposed method in the laboratory experiment is shown in Figure 10a, while the histogram obtained after edge enhancement is shown in Figure 10b. The results of every pixel in the ROI are visualized in its original spatial position as shown in Figure 11a, while the pixels predicted using a frequency of approximately 12.7 Hz are highlighted in Figure 11b. The pixels selected using Canny edge detection with high and low thresholds of 0.2 and 0.08, respectively, which were utilized for edge enhancement, are shown in Figure 11c.

**Figure 12:**
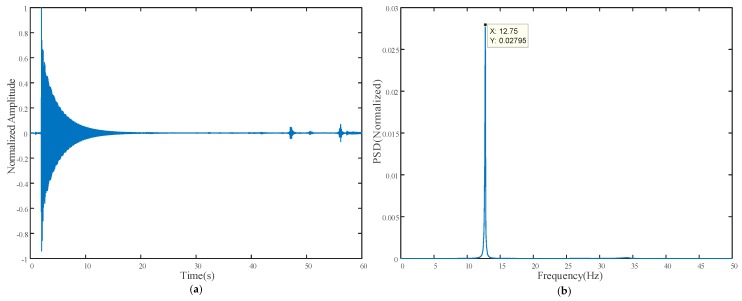
Vibrometer laboratory experimental result: (**a**) Time history of normalized acceleration signal; (**b**) Normalized power spectrum density.

Figure 12 shows the vibration measurement result from the contact vibrometer; the time history of the normalized acceleration signal is plotted in Figure 12a, while the corresponding power spectrum density (PSD) obtained using FFT with peak picking is shown in Figure 12b.

It can be observed that the result obtained from the proposed method closely matched that from the vibrometer, namely 12.4 Hz and 12.75 Hz, respectively. After edge enhancement operation was applied, the noise floor significantly reduced, and a better prediction result of 12.7 Hz was achieved, which is very close to that of the vibrometer. In addition, the highlighted pixels in Figure 11b also demonstrate that the predicted frequency came from the pixels of the vibrometer and carbon plate rather than noise from other parts of the image.

### 3.3. Field Experiment

**Figure 13:**
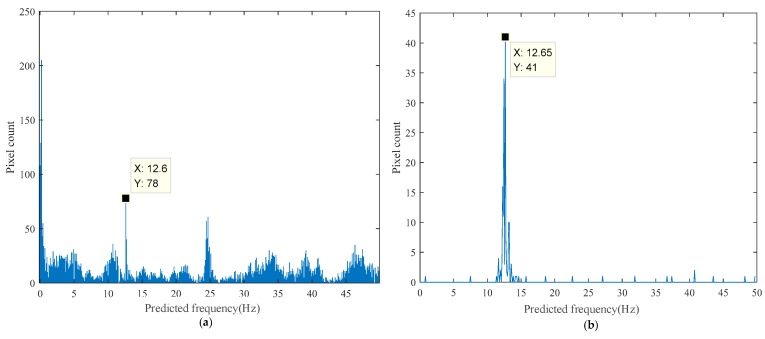
Field experimental results of the proposed method: (**a**) Histogram of predicted frequency distribution; (**b**) Histogram of predicted frequency distribution after edge enhancement.

**Figure 14:**
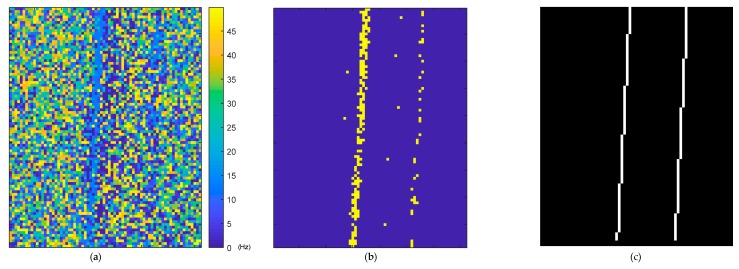
Proposed method field experimental result visualization: (**a**) Prediction result map; (**b**) Pixels predicted around 12.7 Hz (maximum value of histogram) highlighted; (**c**) Pixels used in edge enhancement.

A comparison of the direct prediction result histogram of the field experiment and the corresponding result after edge enhancement is shown in Figure 13. The field experimental result visualization is shown in Figure 14, including the mapped prediction result, while pixels predicted around the maximum value in the histogram are highlighted in Figure 14b. The pixels for the edge enhancement procedure are shown in Figure 14c; the high and low thresholds for the Canny edge algorithm were set to 0.2 and 0.08, respectively.

**Figure 15:**
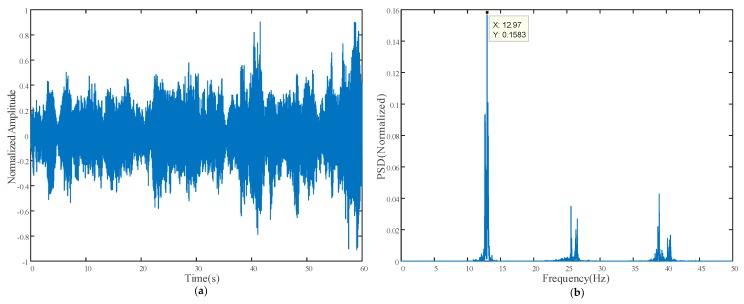
Vibrometer field experimental result: (**a**) Time history of normalized acceleration signal; (**b**) Normalized power spectrum density.

The vibration signal of the bridge cable collected from the vibrometer was normalized and plotted as shown in Figure 15a, while the normalized PSD obtained by FFT is plotted as shown in Figure 15b to examine the frequency components of the bridge cable vibration.

The direct result of the proposed method under field condition, which is challenging for many image-based measurement methods, is shown in Figure 13a. The figure shows significantly higher noise floor than the results obtained from the verification test and laboratory experiment. Many pixels were predicted in the low frequency range under 1 Hz, which may be due to noise from variations in the lighting condition and background of the measurement target, or other forms of disturbance in the image while capturing the video. Excluding the low frequency noise, we can still find the peak with predicted frequency of 12.6 Hz. However, the result with edge enhancement operation shows a clear peak of 12.7 Hz in Figure 13b, and noise was mostly eliminated. The highlighted pixels shown in Figure 14b also confirm that pixels around the measurement object edge are more robust to real-world noise in our method.

The result obtained from the vibrometer was 12.97 Hz, which gives an error of approximately 0.27 Hz (2.1%) compared to the result obtained using the proposed method, that is, 12.7 Hz. One possible explanation for the error is that the vibrometer was installed near the hinge of the bridge cable as this location was convenient and accessible for hand installation as highlighted by the blue circle in Figure 6a, while the ROI for the vibration frequency prediction highlighted by the red rectangle in Figure 6b was significantly higher than the vibrometer installation position to avoid disturbance from passing vehicles and pedestrians in the image.

## 4. Discussion

Based on the experiments conducted with different excitation sources and different test objects vibrating at different frequencies, the following observations were made on the performance of our method. (1) On average, the proposed method can provide acceptable vibration frequency prediction result and the visualized result can improve understanding of the distribution of the vibration frequencies. (2) Under good experimental condition with adequate and stable lighting, as well as good contrast on the measurement target, the proposed method provides acceptable vibration frequency prediction accuracy without any additional operation. Implementing edge enhancement operation improved the predicted result; furthermore, edge enhancement can significantly improve the usability of the predicted result.

Compared to the conventional image-based method, our method does not explicitly analyze the vibration signal to determine the vibration frequency. It uses a single feed-forward CNN to directly predict the vibration frequency. No extra algorithm, image or signal processing technique is required, which makes for a fast, simple, and easy cross-platform deployment. In addition, the CNN model used in the proposed method was trained with purely artificial data, making this method scalable to other frequency ranges or higher precision.

This work also has some limitations. The sampling rate of the input video is restricted to the training configuration; a new model must be trained if a different sampling rate of the input video is to be used. Furthermore, only the peak or resonant frequency is estimated, while other frequency components are not determined. The proposed method is vulnerable to variations in the lighting condition, changes in the background of the measurement target, camera shake, and other forms of electric or mechanical noise.

The method proposed in this paper offers some opportunities for future research. Our method does not require signal preprocessing before feeding into the CNN. A proper signal preprocessing procedure such as signal denoising may improve the performance of the proposed method. The dataset is limited in that it was generated by a simple algorithm and only simple noise was added. The use of a better artificial signal generator that emulates real situations and real noise better will further improve the generalization and robustness of the CNN model in a real-world application.

## 5. Conclusions

In this work, we propose an artificial neural network for vibration frequency prediction. We also propose an optional operation for result enhancement. Experiments were conducted, including verification test, laboratory experiment, and field experiment. A comparison of the results obtained from the proposed method and that of an industrial vibrometer indicates that the proposed method can predict vibration frequency within acceptable error.

The major limitations of this method are that the input video sampling rate is restricted by the trained model, and it cannot estimate all the frequency components of the target. In the future, we will attempt to address these limitations and further research will be conducted focusing on signal preprocessing and dataset preparation for this method.

## Figures and Tables

**Table 1:** Verification test result summary.

Scenario	Excitation Frequency (Hz)	Predicted Frequency (Hz)	
		Direct Result	Edge Enhanced Result
1	5.0	4.9	4.9
2	10.0	10.0	10.0
3	15.0	14.9	14.9
4	20.0	20.1	20.0
5	25.0	25.1	25.1
6	30.0	30.0	30.0
7	35.0	35.0	34.9
8	40.0	40.0	39.9
9	45.0	45.0	45.0
